# 576. Burden of Pneumococcal Disease Due to Serotypes Covered by the 13-Valent and New Higher-Valent Pneumococcal Conjugate Vaccines in All Children and Children at Risk in the United States

**DOI:** 10.1093/ofid/ofac492.628

**Published:** 2022-12-15

**Authors:** Liping Huang, Alejandro D Cane, Johnna Perdrizet, Adriano Arguedas

**Affiliations:** Pfizer Inc, Collegeville, Pennsylvania; Pfizer, Collegeville, Pennsylvania; Pfizer Inc, Collegeville, Pennsylvania; Pfizer, Collegeville, Pennsylvania

## Abstract

**Background:**

Routine vaccination with 13-valent pneumococcal conjugate vaccine (PCV13) in infants along with a catch-up option with PCV13/PPSV23 in children with underlying medical conditions (UMC) have considerably reduced invasive pneumococcal disease (IPD) and non-invasive pneumococcal disease (PD), including community acquired pneumonia (CAP) and acute otitis media (AOM), in the United State (US). However, a rise in IPD and non-invasive PD caused by non-PCV13 serotypes has been observed. A 15-valent PCV (PCV15) and 20-valent PCV (PCV20), containing additional serotypes to PCV13, are anticipated for children soon. The objective of the study was to estimate the annual cases, deaths, and economic burden of disease attributable to PCV13, PCV15, and PCV20 serotypes in children < 18 years old overall and those with UMC in the US.

**Methods:**

Estimated annual cases, deaths and direct medical costs associated with PD caused by PCV13, PCV15 and PCV20 serotypes were calculated based on published incidence rates of IPD, CAP, and AOM, along with age-group specific serotype coverage, case fatality rates, and disease-related costs (Table 1). The PD burden in those with UMC were extrapolated based on incidence rate ratios of those with UMC vs. those without UMC. The results were reported for 0-17 years and were further stratified into 2 age groups: 0-4 and 5-17 years.

**Table 1:**
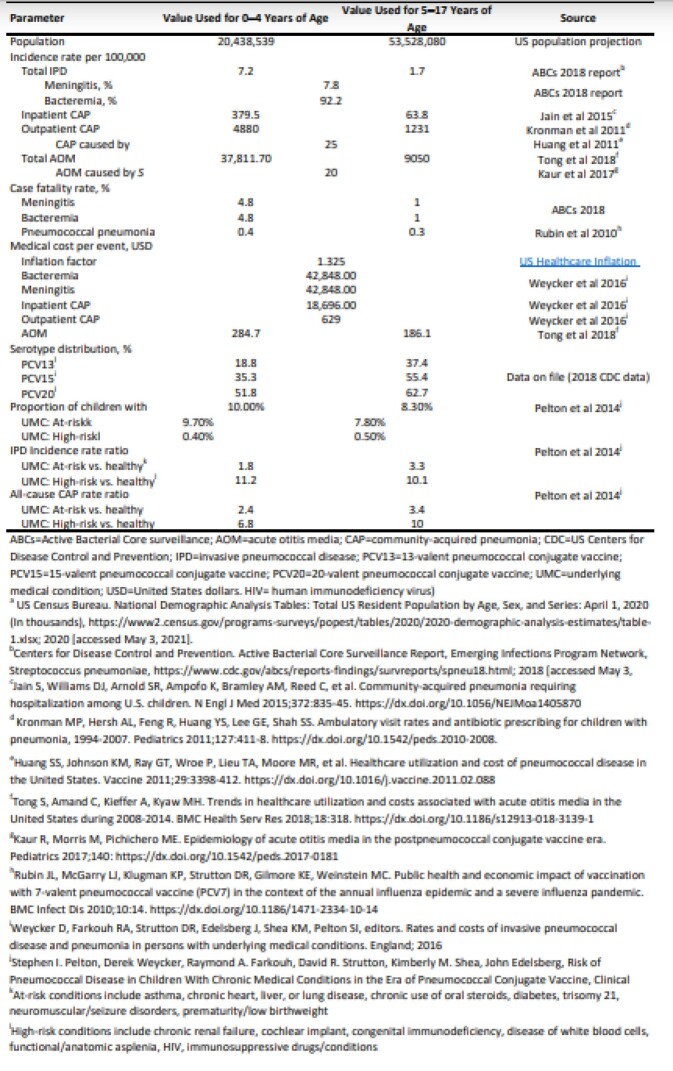
Source Data for the Calculation

**Results:**

The estimated annual PD cases attributable to PCV13, PCV15, and PCV20 serotypes in those 0-17 years were 768,301, 1,275,187, and 1,656,716 and in those with UMC were 23,209, 43,579, 63,949 (Table 2.1), respectively. The estimated direct medical costs were $494, $833, $1,103 million in those 0-17 years, and $76, $127, and $186 million in those with UMC, respectively (Table 3.1). The total estimated IPD cases in children with UMC were 18% (PCV13), 20% (PCV15) and 23% (PCV20) of all estimated IPD cases in all children.

**Figure:**
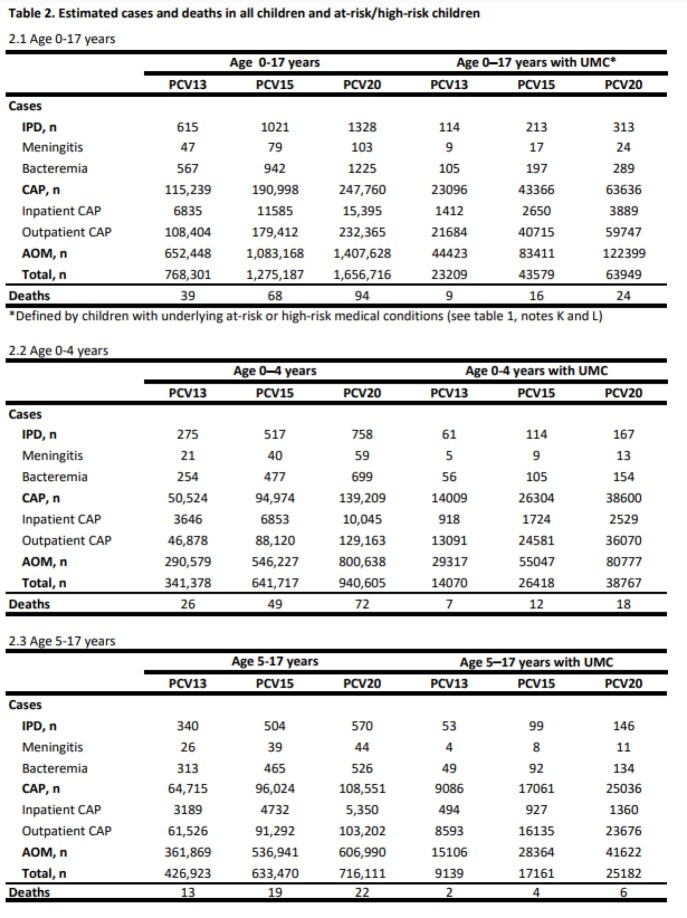

**Figure:**
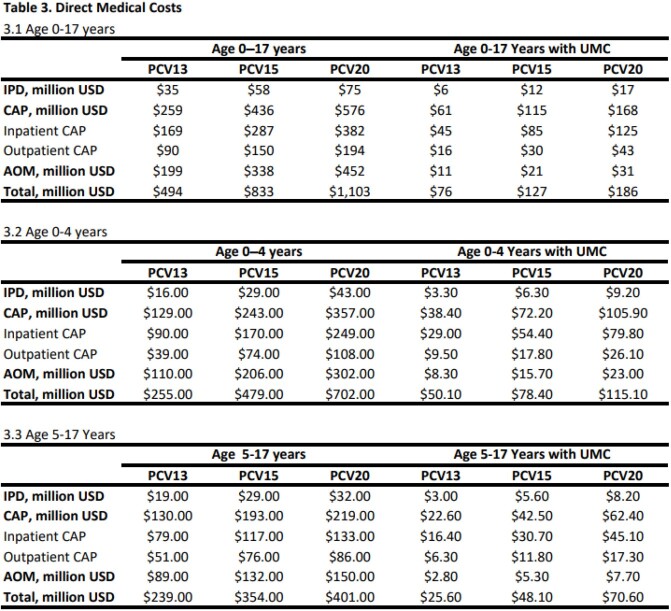

**Conclusion:**

This study demonstrates that the clinical and economic burden associated with new serotypes included in higher valent PCVs are substantial and higher in children with UMCs. The result shows that PCV20 will offer broader PCV coverage in the prevention of PD in ages < 18 years overall and those specifically with UMC.

**Disclosures:**

**Alejandro D. Cane, MD, PhD**, Pfizer: Full time employee|Pfizer: Stocks/Bonds **Johnna Perdrizet, MPH**, Pfizer Inc: Employee|Pfizer Inc: Stocks/Bonds **Adriano Arguedas, Medical director**, Pfizer: Stocks/Bonds.

